# Radioprotective activity of blackcurrant extract evaluated by in vitro micronucleus and gene mutation assays in TK6 human lymphoblastoid cells

**DOI:** 10.1186/s41021-017-0082-z

**Published:** 2017-09-01

**Authors:** Ayumi Yamamoto, Tokuhisa Hirouchi, Saori Kawamorita, Kana Nakashima, Atena Sugiyama, Yoji Kato

**Affiliations:** 1 Chemical and Biological Engineering Course, Department of Industrial System Engineering, National Institute of Technology, Hachinohe College, 16-1 Uwanotai, Tamonoki, Hachinohe-shi, Aomori-ken, 039-1192 Japan; 2 Department of Radiobiology, Institute for Environmental Science, 1-7 Ienomae, Obuchi, Rokkasho-mura, Kamikita-gun, Aomori-ken, 039-3212 Japan; 3 Laboratory of Food Science, Faculty of Education, Hirosaki University, 1 Bunkyo-cho, Hirosaki-shi, Aomori-ken, 036-8560 Japan

**Keywords:** Antigenotoxicity, Antioxidative activity, Genome defense, Radioprotection

## Abstract

**Introduction:**

Blackcurrant (*Ribs nigrum* L.) is a classical fruit that has long been used to prepare juice, jam, liqueur, and sometimes medicines in Europe. Previously, we reported a genome defense effect by the antioxidative activity of several types of blackcurrant extracts (BCEs) in yeast and human cell gene mutation assays. In this study, we determined if BCE exerted radioprotective activity against DNA damage, chromosomal aberration, and gene mutations in the TK6 human lymphoblastoid cell line. We prepared aqueous BCE extracted from mature fruits cultivated in the Aomori Prefecture, Japan.

**Findings:**

In the micronucleus test and TK gene mutation assay, TK6 cells were irradiated with 0, 0.125, 0.250, 0.500, and 1.000 Gy with or without 1.0 mg/mL BCE. Intracellular hydrogen peroxide (H_2_O_2_) was measured using the fluorescent probe BES-H_2_O_2_-Ac. Induction of micronuclei and gene mutations by γ-irradiation exposure was suppressed in combination with BCE. In addition, BCE reduced intracellular H_2_O_2_ levels caused by γ-irradiation.

**Conclusions:**

Our findings clearly support the genome defense potential of blackcurrant against γ-induced DNA damage. We postulate that these genome defense activities are related to the antioxidant compounds in blackcurrant.

## Introduction

Blackcurrant (*Ribes nigrum* L.) is a classical fruit that has long been used to make prepare juice, jam, liqueur, and sometimes medicines in Europe. Blackcurrant, a low deciduous shrub with dark purple fruits containing high levels of polyphenols including anthocyanins, originated in northern Asia and Europe. Currently, Aomori Prefecture in northeast Japan accounts for approximately 90% of blackcurrant production in Japan. The beneficial effects of blackcurrant have been reported worldwide [1].

Recently, we reported that extracts of mature and premature blackcurrant produced in Aomori Prefecture had high anti-oxidant and anti-genotoxic activities, as evaluated using a yeast loss-of-heterozygosity (LOH) assay, an in vitro comet assay to evaluate DNA damage, and a micronucleus (MN) test to screen for genotoxic compounds in human TK6 lymphoblastoid cells [2, 3]. In the yeast LOH assay, blackcurrant extracts (BCEs) extracted from premature and mature fruits suppressed gene mutations induced by hydrogen peroxide (H_2_O_2_), methyl methanesulfonate (MMS), and ultraviolet (UV) radiation [2]. The BCEs showed antigenotoxic effects, with or without heat treatment, against H_2_O_2_-induced oxidative stress in human lymphoblastoid cells, as determined using comet and MN assays [3].

Ionizing radiation, which is a major DNA damaging agent emitted from radioactive substances, induces DNA damage such as oxidative damage and double-stranded breaks (DSBs) [4, 5]. We hypothesized that blackcurrant has the potential to decrease the biological effects of ionizing radiation. To this end, we evaluated the genome defense activity of BCE as a radioprotective agent using a human lymphoblastoid cell line.

## Material and Methods

### Cell culture

The TK6 human lymphoblastoid cell line was grown in RPMI 1640 medium (Nakalai Tesque, Kyoto, Japan) supplemented with 100 U/mL penicillin, 100 μg/mL streptomycin, 0.25 μg/mL amphotericin B, 10% heat-inactivated fetal bovine serum, and 200 μg/mL sodium pyruvate [6]. The cells were incubated at 37 °C in a 5% CO_2_ atmosphere with 100% humidity.

### Chemicals

Trifluorothymidine (TFT) was purchased from Sigma Chemical Company (St. Louis, MO, USA). BES-H_2_O_2_-Ac was purchased from Wako Pure Chemical Industries (Osaka, Japan).

### Irradiation

γ-irradiation was performed using a Pantak HF-320 machine (PANTAK Ltd., East Haven, CT, USA) at 200 kV, 20 mA, and a dose rate of 1.0 Gy/min.

### BCE preparation

Mature blackcurrant fruits, provided from Aomori Blackcurrant Association, Japan, were frozen and preserved until use. The frozen fruit was thawed and mixed with deionized distilled water (DDW) to prepare juice including the soluble components. This blackcurrant solution was sterilized by filtration using the Filtermax rapid vacuum filtration with a 0.22 μm pore size (TPP Techno Plastic Products AG, Trasadingen, Switzerland). After filter sterilization, the solution was freeze-dried and the resulting material was dissolved in DDW at a concentration of 100 mg/mL.

### Combined γ-irradiation exposure and BCE treatment

**Fig. 1:**
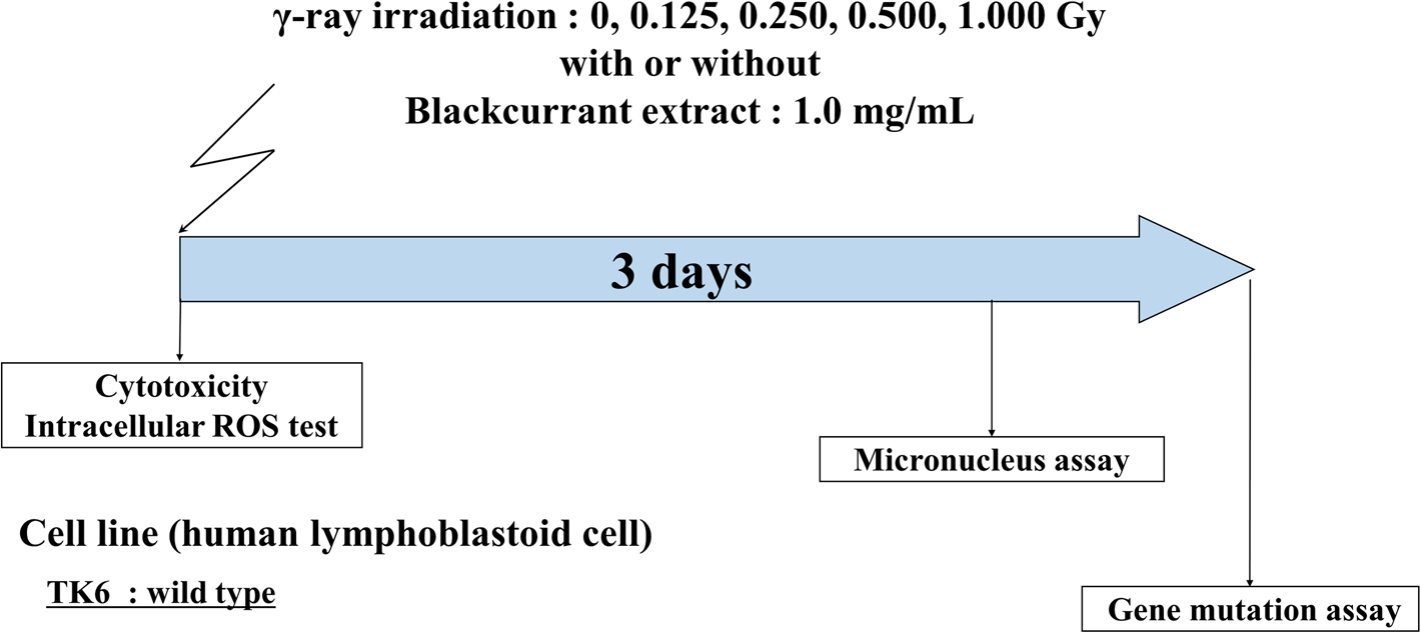
An experimental design to elucidate the combined effects of γ-irradiation and BCE treatment. The TK6 cells were exposed to 0, 0.125, 0.250, 0.500, or 1.000 Gy with or without 1.0 mg/mL BCE. After treatment, cells were immediately subjected to cytotoxicity and intracellular ROS tests. For the MN assay, cells were collected 2 days after treatment. For the gene mutation assay, cells were collected 3 days after the treatment

TK6 was exposed to γ-irradiation at doses of 0.125, 0.250, 0.500, and 1.000 Gy with or without 1.0 mg/mL BCE, a concentration that was decided in accordance with a previous study [3]. The dose rate was 1.0 Gy/min (Fig. 1).

### Genotoxicity assays

Cells exposed to γ-irradiation with or without BCE were incubated for 3 days at 37 °C (Fig. 1), and subsequently collected by centrifugation. TK gene mutation and cytotoxicity assays were performed according to published methods [6, 7]. In the TK gene mutation assay, TK6 cells were plated at a density of 40,000 cells/well with 3.0 μg/mL TFT. In the cytotoxicity assay, cells were immediately seeded at a density of 1.6 cells/well in 96-well plates after irradiation with or without BCE. All plates were incubated at 37 °C in a humidified atmosphere of 5% CO_2_. Normal-growing (NG) colonies after incubation for 2 weeks and slow-growing (SG) colonies after incubation for 4 weeks were counted on the mutation assay plates containing TFT. The MN assay was performed as previously described [3, 7, 8]. Briefly, after 48 h of irradiation, approximately 10^6^ cells were suspended in 0.075 M potassium chloride and then incubated for 10 min at room temperature. The suspended cells were fixed in ice-cold methanol containing 25% acetic acid, followed by centrifugation, re-suspension in ice-cold methanol containing 25% acetic acid, and suspension in ice-cold methanol containing 1.0% acetic acid. A drop of fixing cell solution was spotted onto glass slides, which were then air-dried. The fixed cells were stained with acridine orange (Wako Pure Chemical Industries) and analyzed with a fluorescence microscope (Olympus, Tokyo, Japan).

### Measurements of intracellular reactive oxygen species

The levels of intracellular reactive oxygen species (ROS) were measured using 50 μM BES-H_2_O_2_-Ac fluorescence probe purchased from Wako Pure Chemical Industries. Then 5 × 10^5^ cells/mL of TK6 cells were incubated with BES–H_2_O_2_–Ac for 1 h. After washing with phosphate-buffered saline, TK6 cells were exposed to γ-irradiation with or without BCE. More then 100 cells were counted to estimate the percentage of ROS-positive cells. The increase in the proportion of fluorescent cells was calculated by comparison with control cells, which lacked BCE treatment and γ-irradiation.

### Statistical analysis

For all of the assays, statistical analyses were performed using the Student’s *t*-test. The standard deviation was calculated from three separate experiments. Data analyses were performed with Microsoft Excel 2016 software (Microsoft, Redmond, WA, USA).

## Results

### Inhibition of γ-irradiation-induced MN formation by treatment of TK6 cells with BCE

**Fig. 2:**
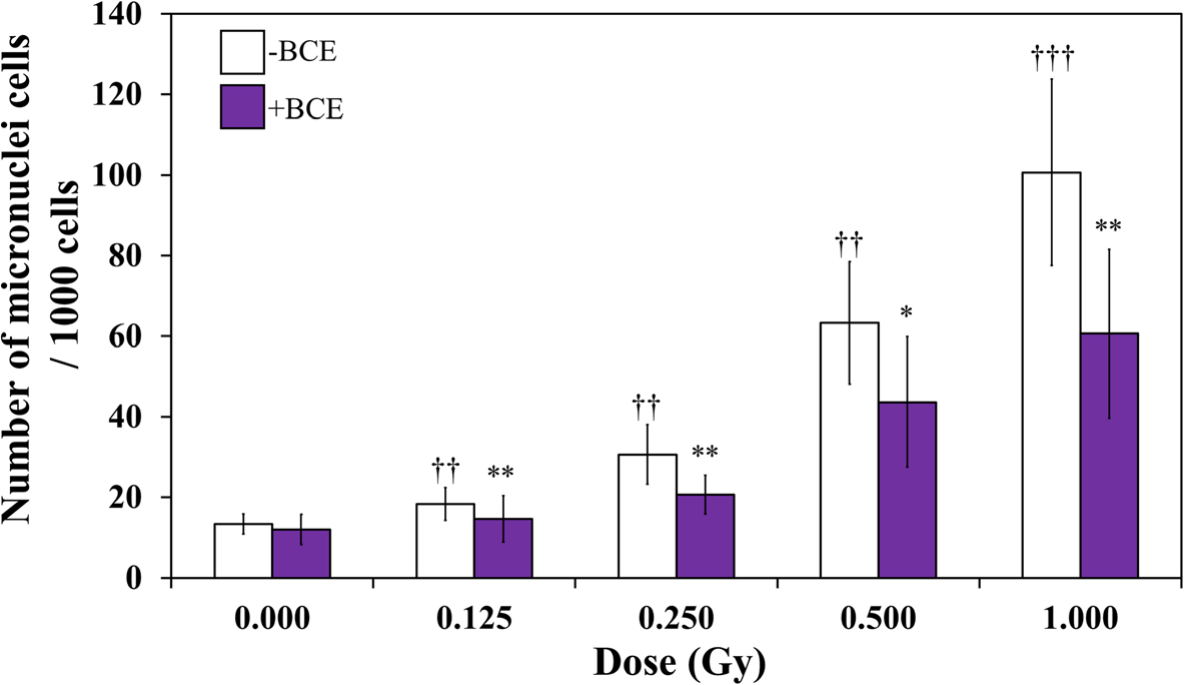
MN assay in TK6 cells. Daggers (††) and daggers (†††) denote *p* < 0.05 and *p* < 0.01, respectively, in the *t*-test comparing control and γ-irradiated cells. An asterisk (*) and asterisks (**) denote *p* < 0.1 and *p* < 0.05, respectively, in the *t*-test of comparing BCE-untreated and BCE-treated cells

Treatment with γ-irradiation alone increased the frequency of MN formation, determined y an increase in the number of cells with MN per 1000 TK6 cells (Fig. 2). The γ-irradiated cells showed significantly higher MN frequencies than non-irradiated cells without BCE treatment. With combined treatment, MN frequencies were significantly decreased when the cells were treated with 1.0 mg/mL BCE at γ-irradiation doses of 0.125, 0.250, 0.500, and 1.000 Gy (*p* < 0.05, *p* < 0.05, *p* < 0.1, and *p* < 0.05, respectively). In addition, with 1.0 mg/mL BCE, decreases in MN by 12 ± 13%, 24 ± 16%, 32 ± 8%, 32 ± 15%, and 40 ± 13% were obtained at *γ*-irradiation doses of 0.125, 0.250, 0.500, and 1.000 Gy, respectively. These results suggest that treatment with 1.0 mg/mL BCE exerts suppressive effects on *γ*-induced MN formation in TK6 cells.

### Suppressive effects of cytotoxicity and mutagenicity of γ-irradiation with combined treatment of BCE and γ-irradiation in TK6 cells

**Fig. 3:**
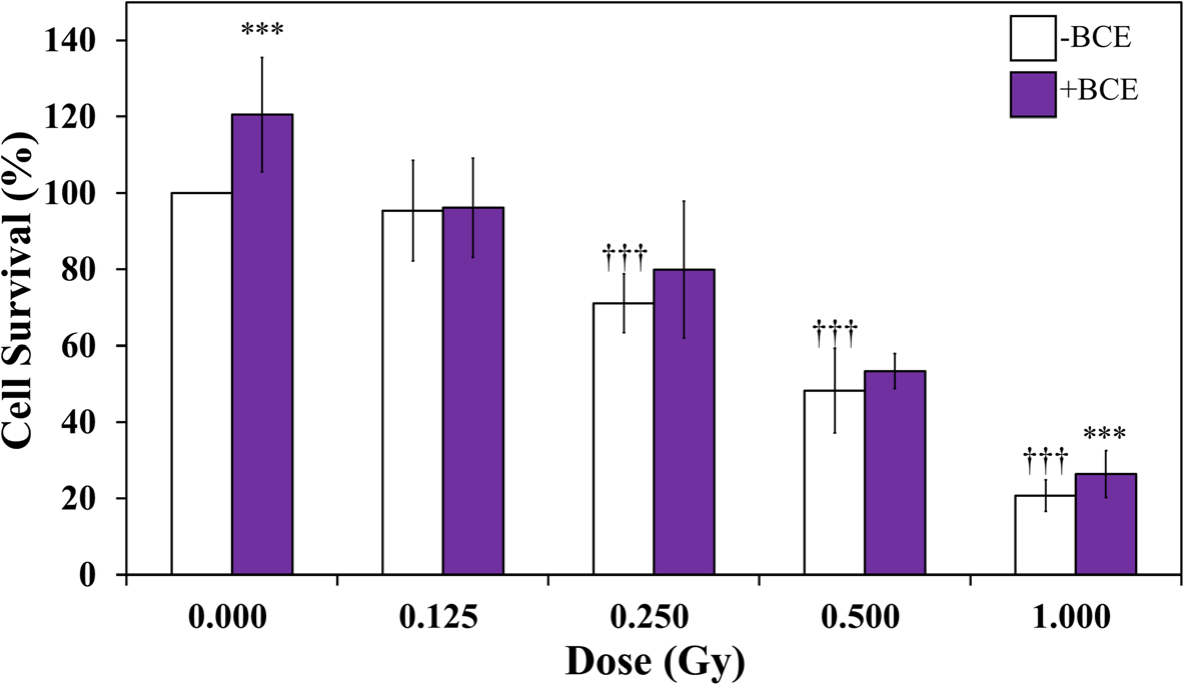
Effects of irradiation on the survival of TK6 cells. Daggers (†††) denote *p* < 0.01 in the *t*-test comparing control and γ-irradiated cells. Asterisks (***) denote *p* < 0.01 in the *t*-test comparing BCE-untreated and BCE-treated cells

**Fig. 4:**
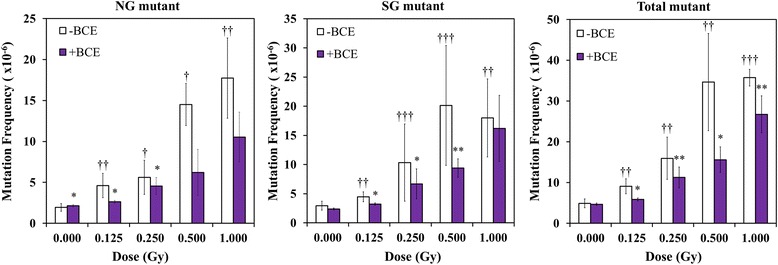
TK gene mutation assay in TK6 cells. Daggers (††) and daggers (†††) denote *p* < 0.05 and *p* < 0.01, respectively, in the *t*-test comparing control and γ-irradiated cells. An asterisk (*) and asterisks (**) denote *p* < 0.1 and *p* < 0.05, respectively, in the *t*-test comparing BCE-untreated and BCE-treated cells

Exposure of TK6 cells to γ-irradiation without BCE treatment at doses of 0.125, 0.250, 0.500, and 1.000 Gy resulted in 95.4%, 71.1% (*p* < 0.01), 48.3% (*p* < 0.01), and 20.8% (*p* < 0.01) cell survival, respectively (Fig. 3). Cell survival decreased with γ-irradiation. The survival of TK6 cells treated with BCE at radiation doses of 0.000 and 1.000 Gy was significantly higher than that of cells without BCE treatment (*p* < 0.01). These results suggest that the combined treatment of BCE at a concentration of 1.0 mg/mL exerts a suppressive effect on the cell-killing effect of γ-irradiation. TK6 generates two distinct phenotypic classes of TK mutants. NG mutants have the same doubling time as wild-type cells, and the doubling time of SG mutants is longer than that of wild-type and NG mutants. NG mutants contain small DNA mutations such as point mutations, small insertions, and small deletions, whereas SG mutants contain large chromosomal structure changes. In the mutation assay, γ-irradiation without BCE treatment enhanced NG and SG TK mutation frequencies of TK6 cells with significant differences (Fig. 4, Table 1). γ-irradiation alone induced total mutations (tNG and SG mutations) at rates of 9.08 × 10^−6^, 15.95 × 10^−6^, 34.64 × 10^−6^ and 35.74 × 10^−6^ TK6 cells exposed to 0.125, 0.250, 5.000, and 1.000 Gy, respectively (*p* < 0.05, *p* < 0.05, *p* < 0.01, and *p* < 0.01, respectively). In the combination treatment with 1.0 mg/mL BCE and γ-irradiation, total mutation frequencies decreased when the cells were treated with 1.0 mg/mL BCE at all four doses (*p* < 0.1, *p* < 0.05, *p* < 0.1, and *p* < 0.05, respectively). The combined treatment with BCE suppressed NG mutation frequencies at doses of 0.000, 0.125, and 0.250 Gy with marginal significance (*p* < 0.1, *p* < 0.1, and *p* < 0.1, respectively). SG mutation frequencies at doses of 0.125, 0.250, and 0.500 Gy were reduced by BCE combination treatment with significant and marginally significant differences (*p* < 0.1, *p* < 0.1 and *p* < 0.05, respectively). These results suggest that the combined treatment of BCE and γ-irradiation exerts a suppressive effect against several types of γ-induced gene mutations.

**Table 1:** Results of TK gene mutation assay in TK6 cells

Irradiation dose (Gy)	0.000	0.125	0.250	0.500	1.000
Mutant Frequency (×10^−6^ cells)					
BCE untreated					
NG mutant	2.95 ± 0.76	4.48 ± 0.84 (*p* < 0.05)^††^	10.33 ± 6.62 (*p* < 0.1)^†^	20.13 ± 10.28 (*p* < 0.1)^†^	18.00 ± 6.68 (*p* < 0.05)^††^
SG mutant	1.95 ± 0.470.47	4.61 ± 1.48 (*p* < 0.05)^††^	5.62 ± 2.09 (*p* < 0.01)^†††^	14.51 ± 2.57 (*p* < 0.01)^†††^	17.74 ± 4.91 (*p* < 0.05)^††^
Total mutant	4.90 ± 1.07	9.08 ± 1.84 (*p* < 0.05)^††^	15.95 ± 5.21 (*p* < 0.05)^††^	34.64 ± 11.94 (*p* < 0.05)^††^	35.74 ± 2.04 (*p* < 0.01)^†††^
BCE treated					
NG mutant	2.38 ± 0.15	3.22 ± 0.21 (*p* < 0.1)*	6.69 ± 2.57 (*p* < 0.1)*	9.40 ± 1.59	16.20 ± 5.67
SG mutant	2.14 ± 0.13	2.63 ± 0.17 (*p* < 0.1)*	4.56 ± 1.04 (*p* < 0.1)*	6.21 ± 2.82 (*p* < 0.05)**	10.54 ± 3.05
Total mutant	4.68 ± 0.27	5.85 ± 0.38 (*p* < 0.1)*	11.25 ± 2.58 (*p* < 0.05)**	15.61 ± 3.13 (*p* < 0.1)*	26.74 ± 4.55 (*p* < 0.05)**

Dagger^†^, daggers^††^ and daggers^†††^ denote *p* < 0.1, *p* < 0.05 and *p* < 0.01, respectively, in the *t*-test comparing non-irradiated and γ-irradiated cells. An asterisk^*^ and asterisks^**^ denote *p* < 0.1 and *p* < 0.05, respectively, in the *t*-test of comparing BCE-untreated and BCE-treated cells

### Scavenging activity of γ-induced intracellular ROS by combined treatment with BCE in TK6 cells

**Fig. 5:**
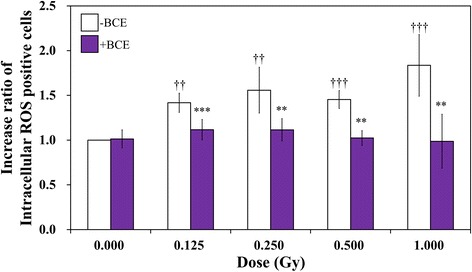
Intracellular ROS (H_2_O_2_) assay in TK6 cells. Daggers (††) and daggers (†††) denote *p* < 0.05 and *p* < 0.01, respectively, in the *t*-test comparing control and γ-irradiated cells. Asterisks (**) and asterisks (***) denote *p* < 0.05 and *p* < 0.01, respectively, in the *t*-test comparing BCE-untreated and treated cells

Because our previous study found that BCE has antioxidant activity, we focused on intracellular ROS in BCE-treated TK6. H_2_O_2_ is a marker of oxidative stress and is generated by radiation [9]. Thus, we assayed intracellular H_2_O_2_ levels as an index of ROS to evaluate the scavenging activity of BCE against γ-induced ROS using the fluorescent probe BES-H_2_O_2_-Ac, a highly specific H_2_O_2_ indicator. γ-irradiation induced a higher level of intracellular ROS than nonirradiated cells without BCE treatment (Fig. 5). The increasing rates of γ-irradiated cells were 1.42 ± 0.11, 1.56 ± 0.26, 1.45 ± 0.10, and 1.84 ± 0.34 obtained from 0.125, 0.250, 0.500, and 1.000 Gy (*p* < 0.05, *p* < 0.05, *p* < 0.01, and *p* < 0.01, respectively). In contrast, upon combined treatment, the increasing rate of intracellular ROS was 1.01 ± 0.10, 1.12 ± 0.11, 1.11 ± 0.12, 1.02 ± 0.08, and 0.99 ± 0.30 for the same *γ*-irradiation doses. There were significant differences between BCE-untreated and BCE-treated cells. The *p* values of the TK6 cells γ-irradiated at 0.125, 0.250, 0.500, and 1.000 Gy were *p* < 0.01, *p* < 0.05, *p* < 0.05, and *p* < 0.05, respectively. BCE-treated cells suppressed the intracellular ROS induced by γ-irradiation with significant differences from untreated cells.

## Discussion

Our observations that BCE suppressed not only radiation-induced MN and gene mutations but also radiation-induced intracellular ROS indicate that BCE has radioprotective activity.

Radiation induces DSBs in DNA, which result in chromosomal aberrations and cell death, generating a wide variety of ROS that induce gene mutations following the occurrence of oxidative bases in DNA [10]. The production of DSBs in DNA activates DNA damage checkpoint signaling and DNA repair pathways, which employ nonhomologous end joining (NHEJ) and homologous recombination (HR) [11]. In contrast, ionizing radiation induces ROS including superoxide anions, hydrogen peroxide, and hydroxyl radicals [12]. These ROS damage several kinds of biomolecules including DNA, proteins, and lipids [13]. ROS is scavenged by superoxide dismutase and catalase [13, 14]. Oxidative nucleotides such as 8-hydroxyguanosine (8-oxodG, 8OHdG, and 8OHG) are mutagenic lesions that are formed by ROS in the nucleotide pool as well as in DNA [15–18].

Previously, we reported the antigenotoxic effects of BCE evaluated using yeast LOH assay and human lymphoblastoid cell assays [2, 3]. In a yeast gene mutation assay, BCEs from premature and mature fruit, inhibited DNA mutations induced by H_2_O_2_, methyl MMS as an alkylating agent, and UV as physical stress [2]. Several types of BCEs extracted from mature and premature fruit with or without heat treatment exhibited antigenotoxic and antioxidative activities against H_2_O_2_-induced DNA damage and intracellular ROS in TK6 cells [3]. BCE contains high concentrations of polyphenolic compound, including anthocyanin, and of L-ascorbic (vitamin C) acid, which exerts antioxidant activity with radical scavenging [2, 3]. We evaluated the radical scavenging activity of polyphenolic compounds such as anthocyanins and catechin. BCE-containing anthocyanins and vitamin C induce small structural changes in erythrocyte membranes that exert beneficial effects on protection against oxidation [19]. There is a possibility that cell membrane modifications were involved in the radioprotective activity of BCE observed in this study.

Recently, Nishimura et al. [20] reported that *Actinidia argute*, known as sarunashi, has antimutagenic and antioxidant activities. Furthermore, an extract of Barbados cherry fruit containing several antioxidants such as anthocyanin, vitamins E and C, and β-carotene showed antimutagenic effects, as measured by a MN test against radioisotope iodine-131, and this antimutagenic activity was mainly associated with the capture of free radicals generated by radiation [21, 22]. Polyphenols showed radioprotective properties associated with their high antioxidant activity in in vitro and in vivo assays [23–29]. Our findings are consistent with those in these reports. Dietary polyphenols and their analogs such as resveratrol, curcumin, quercetin, and catechin may activate the expression of DNA repair proteins such as the NHEJ and HR proteins Ku70, NBS, Werner helicase, and Nijmegen Breakage Syndrome protein (nibrin) through the promotion of the class III histone deacetylase sirtuin1 (SIRT1) [30]. SIRT1 is associated with the DNA damage response to γ-irradiation and is directly associated with DSB repair proteins [31]. An anthocyanin, D3R, upregulated SIRT1 expression in human umbilical vein endothelial cells [32]. In our experiments, micronuclei in the MN test and the SG mutation in the TK gene mutation assay, which are associated with DSBs caused by irradiation, were suppressed by BCE treatment. We speculate that BCE anthocyanins upregulate SIRT1 expression and modify the activity of HR-associated proteins. However, while ROS, micronuclei, and gene mutations induced by γ-irradiation were strongly inhibited by the combined treatment of BCE and γ-irradiation, BCE treatment did not cause complete recovery of cell survival. Future studies are needed to clarify the mechanism that underlies this radioprotective effect according to different activities and constituents of polyphenols and other antioxidants, as well as the interaction between these compounds and DNA repair mechanisms. In this study, we treated TK6 cells concurrently with BCE and γ-irradiation. Thus, future studies should evaluate the optimal timing and best concentration of BCE to use for therapeutic purposes.

In conclusion, we demonstrated that BCE exerted genome defense activities against γ-irradiation-induced micronuclei, gene mutations, and oxidative stress in the TK6 human lymphoblastoid cell line. Because oxidative stress is one of the major factors in γ-radiation-induced genome instability, which is associated with carcinogenesis and several diseases, we suggest that BCE activities may protect cells from damage induced by γ-irradiation. This is the first study to show the radioprotective activity of BCE.

## Abbreviations

BCE: The extract obtained from the fruit of blackcurrant
C3G: Cyanidin 3-*O*-β-glucoside
C3R: Cyanidin 3-*O*-β-rutinoside
D3G: Delphinidin 3-*O*-β-glucoside
D3R: Delphinidin 3-*O*-β-rutinoside
DDW: Deionized distilled water
DSBs: Double stranded breaks
HDAC: Histone deacetylase
HR: Homologous recombination
LOH: Loss of heterozygosity
MMS: Methylmethanesulfonate
MN: Micronucleus
NG: Normally growing
NHEJ: Nonhomologous end joining
PBS: Phosphate buffered saline
ROS: Reactive oxygen species
SG: Slowly growing
SIRT1: Sirtuin 1
TFT: Trifluorothymidine
UV: Ultraviolet
